# Hydrogen Production from Ammonia Decomposition: A Mini-Review of Metal Oxide-Based Catalysts

**DOI:** 10.3390/molecules29163817

**Published:** 2024-08-12

**Authors:** Senliang Xi, Wenying Wu, Wenhao Yao, Ruodan Han, Sha He, Wenju Wang, Teng Zhang, Liang Yu

**Affiliations:** 1 Key Laboratory of Cluster Science Ministry of Education, Beijing Key Laboratory of Photoelectronic/Electrophotonic Conversion Materials, Advanced Research Institute of Multidisciplinary Science, School of Chemistry and Chemical Engineering, Beijing Institute of Technology, Beijing 100081, China; xisenliang1996@163.com (S.X.); hanruodan@126.com (R.H.); 2 Advanced Technology Research Institute (Jinan), Beijing Institute of Technology, Jinan 250000, China; hesha335611003@163.com; 3 School of Energy and Power Engineering, Nanjing University of Science and Technology, Nanjing 210094, China

**Keywords:** hydrogen storage, catalyst, ammonia, metal oxides

## Abstract

Efficient hydrogen storage and transportation are crucial for the sustainable development of human society. Ammonia, with a hydrogen storage density of up to 17.6 wt%, is considered an ideal energy carrier for large-scale hydrogen storage and has great potential for development and application in the “hydrogen economy”. However, achieving ammonia decomposition to hydrogen under mild conditions is challenging, and therefore, the development of suitable catalysts is essential. Metal oxide-based catalysts are commonly used in the industry. This paper presents a comprehensive review of single and composite metal oxide catalysts for ammonia decomposition catalysis. The focus is on analyzing the conformational relationships and interactions between metal oxide carriers and active metal sites. The aim is to develop new and efficient metal oxide-based catalysts for large-scale green ammonia decomposition.

## 1. Introduction

Hydrogen is an ideal fuel that burns without pollutant emissions and has the highest calorific value (142 MJ kg^−1^) of any fuel. With the use of renewable energy sources and the commercialization of fuel cell systems, hydrogen is gaining popularity as a promising energy carrier. However, hydrogen has a low density and low boiling point, and it is difficult to liquefy, making it extremely inefficient, expensive, and unsafe to transport and store at high pressure or low temperature in transport vehicles. Therefore, hydrogen storage is a critical challenge for future hydrogen applications in fuel cell systems [1,2]. Compared to high-pressure gaseous hydrogen storage and low-temperature liquid hydrogen storage, physical adsorption storage materials, such as porous carbon material [3,4] and metal–organic frameworks (MOFs) [5,6,7,8], etc., and chemical hydrogen storage materials, such as NaBH_4_ [9,10,11,12], MgH_2_ [13,14,15,16,17], NH_3_BH_3_ [18,19,20] and NH_3_ [21,22,23,24,25], have the advantage of efficiency, safety, and high storage efficiency. These can produce hydrogen in situ after undergoing desorption, reaction, or decomposition.

Among the many candidates, ammonia has a high volumetric hydrogen storage density (108 kg H_2_ m^−3^ NH_3_ liquid) and gravimetric hydrogen storage density (17.8 wt%) and is liquefied at 20 °C under 8.6 bar; moreover, as a basic chemical material, the process of ammonia synthesis is very mature, so ammonia is one of the most promising carriers of hydrogen energy [26,27,28,29]. Moreover, The Global Ammonia Energy Alliance proposed the new concept of Ammonia = Hydrogen 2.0 in 2017. Therefore, creating a new system of green ammonia energy is beneficial to promote the development of hydrogen energy. The process of hydrogen production from the decomposition of ammonia requires the absorption of a large amount of heat. Above 200 °C, ammonia begins to decompose into hydrogen and nitrogen, but complete conversion takes place above 650 °C. The thermal decomposition of ammonia is a two-step process involving the breaking of the N-H bond in one step and the recombination and desorption of nitrogen atoms and hydrogen atoms into the N_2_ and H_2_ molecules, respectively [30]. Furthermore, hydrogen production from ammonia decomposition is a process that does not emit CO_x_, which is especially important for fuel cells operating at low temperatures such as polymer electrolyte fuel cells, where the presence of CO can severely degrade the performance of the platinum electrode. The efficient generation of hydrogen from ammonia is key to its use as a hydrogen energy carrier. Ammonia decomposition is a heat-absorbing reaction, and the ammonia decomposition equation calculates that the ammonia conversion is as high as 99.11% at 1 atm under 400 °C [31]. However, the slow kinetic properties of the ammonia decomposition process cause the ammonia decomposition to require an increase in temperature to improve the efficiency of ammonia decomposition, and it usually requires more than 650 °C to fully convert the ammonia, which is too high a temperature for existing hydrogen fuel cells. Therefore, the development of efficient catalysts to improve the kinetic property of hydrogen production from ammonia decomposition to achieve complete ammonia decomposition at low temperatures is crucial to promote the application of hydrogen fuel cells in the life of human society.

Recently, researchers have synthesized efficient metal-loaded catalysts for hydrogen production from ammonia decomposition by controlling the size, shape, and surface state of metal nanoparticles using different supports, such as carbon materials [32,33,34], metal nitrides [35,36,37] and metal oxides [38,39,40]. Metal oxide carrier catalysts are typical of non-homogeneous catalytic systems. Typically, metal nanoparticles are the main catalytically active phase, while oxide carriers provide such effects as stabilization of the nanoparticles, construction of a high specific surface, abundant defect sites, and acidic or basic site donors. Furthermore, the high activity of the metal site results in a robust adsorption effect on the H atoms following the dissociation of NH_3_. This can readily lead to the occupation of the metal active center by H atoms, thereby triggering the phenomenon of H poisoning and consequently causing deactivation of the catalyst. However, the hydrogen overflow phenomenon observed on the surface of metal oxides effectively circumvents the poisoning of the catalyst [41].

**Scheme 1:**
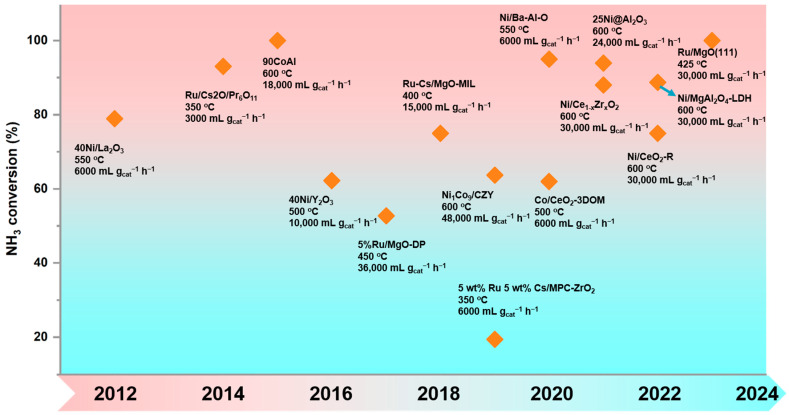
Summary of recent developments of metal oxide-based catalysts for the ammonia decomposition.

In this review, we summarized oxide-based ammonia decomposition catalysts (Scheme 1) and analyzed the interaction between oxides and loaded metals. The prospects for the development of high-performance ammonia decomposition catalysts are presented.

## 2. Single Metal Oxide Supports

Over the past decade, a large amount of research work has revolved around the use of monometallic oxides as carriers for loading metal nanoparticles as catalysts to improve the performance of ammonia decomposition, such as Al_2_O_3_ [42,43,44,45,46,47], MgO [48,49,50] and rare-earth metal oxides such as CeO_2_ [51,52,53,54,55,56,57,58], Pr_2_O_3_ [59], Sm_2_O_3_ [60] and La_2_O_3_ [61].

### 2.1. Al_2_O_3_ Support

Al_2_O_3_ is a commonly used carrier material for industrial catalysts, which has the advantages of good mechanical strength, good thermal and chemical stability, and variable surface acidity and alkalinity. A large amount of work has been carried out around Al_2_O_3_ support. In 2009, Vlachos et al. [62] prepared a series of Ru nanoparticles of different sizes and shapes by loading the noble metal Ru onto alumina and varying the reduction temperature as well as the calcination temperature and investigated the effect of particle size and shape on the ammonia decomposition activity of Ru/γ-Al_2_O_3_ catalysts. The constructed microkinetic models demonstrate that the performance of ammonia decomposition does not necessarily improve with decreasing nanoparticle size. As the size of the Ru nanoparticles becomes larger, the shape gradually changes from round to flat and then to large elongated particles, and the terraced structure in the large elongated particles shape enables the Ru nanoparticles to form more B5 active sites, thus leading to the enhancement of the ammonia decomposition performance.

In addition to improving the catalytic activity for ammonia decomposition by adjusting the shape and particle size of Ru nanoparticles on Al_2_O_3_, different catalytic activities have been demonstrated by changing the crystalline phase of the Al_2_O_3_ carriers to regulate the interactions between the Al_2_O_3_ and the Ru nanoparticles. Kim and Park [63] used six different crystalline alumina types (α-Al_2_O_3_, κ-Al_2_O_3_, θ-Al_2_O_3_, δ-Al_2_O_3_, η-Al_2_O_3_, and γ-Al_2_O_3_) as the carriers and prepared a series of Ru-based catalytic substrates by wet impregnation to study the effect of different crystalline types of carriers on the performance of Ru-based catalysts for ammonia decomposition. The sizes of Ru nanoparticles corresponding to Ru/α-Al_2_O_3_, Ru/κ-Al_2_O_3_, Ru/θ-Al_2_O_3_, Ru/δ-Al_2_O_3_, Ru/η-Al_2_O_3_ and Ru/γ-Al_2_O_3_ after calcination at 100 °C were 4.2 ± 1.2, 3.0 ± 0.9, 2.6 ± 0.5, 2.1 ± 0.4, 1.8 ± 0.2 and 1.3 ± 0.4 nm, respectively. Among them, Ru/α-Al_2_O_3_ and Ru/κ-Al_2_O_3_ with larger Ru particle sizes showed relatively better catalytic properties. This reflects the differences in the interaction of different crystalline alumina carriers for Ru nanoparticles, leading to differences in particle size during wet impregnation as well as reduction. When calcined at 300 °C, the sizes of Ru nanoparticles corresponding to Ru/α-Al_2_O_3_, Ru/κ-Al_2_O_3_, Ru/θ-Al_2_O_3_, Ru/δ-Al_2_O_3_, Ru/η-Al_2_O_3_ and Ru/γ-Al_2_O_3_ were 8.4 ± 2.3, 8.2 ± 2.0, 9.8 ± 1.9, 11 ± 2, 7.3 ± 1.3 and 7.7 ± 2.1 nm, respectively. Despite the larger size of the Ru nanoparticles after high-temperature sintering, there will still be several carriers (κ-Al_2_O_3_, θ-Al_2_O_3_, δ-Al_2_O_3_, η-Al_2_O_3_, and γ-Al_2_O_3_) with improved catalytic properties. Not only does this corroborate the point made by Vlachos et al. [62], but it also shows the difference in crystalline shape for the catalytic process. Among this series of catalysts, Ru/κ-Al_2_O_3_(C300) has the fastest hydrogen production rate, reaching 0.7 mol_H2_ (g_Ru_ min)^−1^.

**Figure 1:**
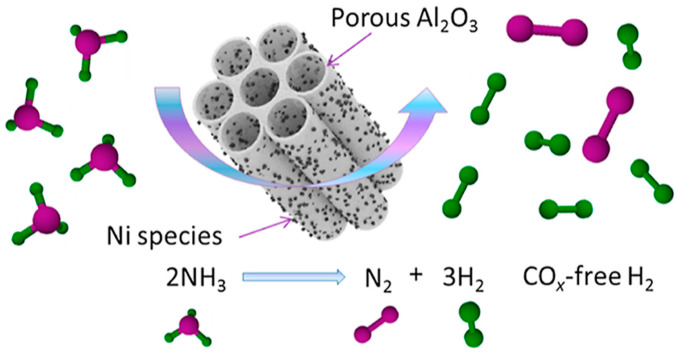
Schematic representation of a catalyst (Ni@Al_2_O_3_) synthesized by a simple one-pot method with homogeneous nickel particles of small size [42].

Although the catalytic performance of non-precious metals is not as good as that of Ru in terms of intrinsic activity, the performance of ammonia decomposition can be further enhanced by changing the structure of the carrier or changing the structure between the active metals. Wang and Zhang et al. [42] prepared mesoporous NiO@Al_2_O_3_ catalysts by the soft template method as shown in Figure 1 and varied the loading of active metal Ni by changing the ratio between Ni and Al. During ammonia decomposition, the active site NiO becomes Ni nanoparticles by in situ reduction, and the (111) crystalline surface of Ni is observed. Moreover, the size as well as the distribution of Ni nanoparticles reduced in situ by NiO varied due to the different content of loaded Ni. This results in more adsorption of the active site Ni with NH_3_ in the catalytic process. Among them, the conversion of ammonia reached 93.9% for 25Ni@Al_2_O_3_ at 600 °C under 24,000 mL g_cat_^−1^ h^−1^, and the catalytic performance remained stable within 24 h. This is mainly attributed to the fact that the porous property of Al_2_O_3_ can effectively inhibit the sintering of Ni during the high-temperature catalytic process. Murciano et al. [43] used adsorption, impregnation, and precipitation methods to load Co onto Al_2_O_3_ carriers. From the results, it was shown that the Co nanoparticles obtained from the catalysts precipitated using carbonate buffer had a small size, while the Co species were more highly reduced, allowing them to contain more catalytic sites, thus providing superior performance compared to catalysts prepared by other methods.

### 2.2. MgO Support

**Figure 2:**
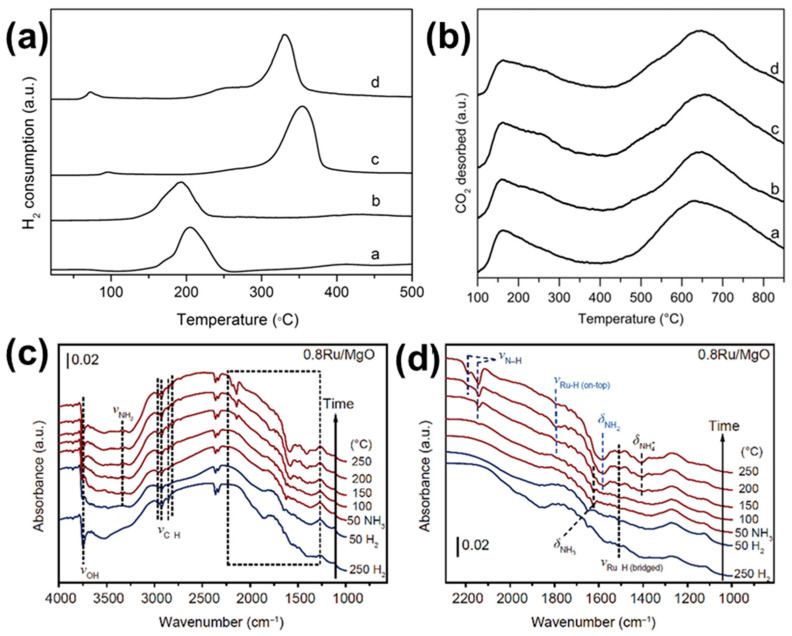
(**a**) TPR profiles of Ru/MgO catalysts: a 3%Ru/MgO-IM; b 5%Ru/MgO-IM; c 3%Ru/MgO-DP; d 5%Ru/MgO-DP [48]. (**b**) CO_2_-TPD spectra of reduced a 5%Ru/MgO-DP, b 1%Ru/c-MgO-DP, c 3%Ru/c-MgO-DP, and d 5%Ru/c-MgO-DP [49]. (**c**) In situ DRIFTS study of NH_3_ adsorption on 0.8Ru/MgO during the heating process; (**d**) the partial enlarged detail of (**c**) in the dashed box (color online) [64].

MgO is a basic oxide with excellent resistance to sintering, making it an excellent carrier for ammonia decomposition catalysts. Ping Chen’s group [48] prepared a class of mesoporous Ru/MgO catalysts by the deposition precipitation method, which greatly improved the dispersion of Ru nanoparticles compared with the conventional wet chemical impregnation method. Meanwhile, it was found by the H_2_-TPR test (Figure 2a) that the catalysts prepared by the deposition precipitation method could be reduced above 300 °C, while the catalysts prepared by the wet chemical impregnation method could be reduced below 200 °C, which indicated that the Ru/MgO-DP had better thermal stability than the Ru/MgO-IM. By testing the ammonia decomposition performance, the ammonia conversion of Ru/MgO-DP prepared by the deposition precipitation method reached 56.5% at 450 °C with a temporal rate of 30,000 mL g_cat_^−1^ h^−1^, which was much higher than that of Ru/MgO-IM prepared by the wet chemical impregnation method (less than 20%). Based on this work, Chen et al. [49] further investigated the effect of alkaline sites on the surface of the carrier MgO on the ammonia decomposition performance. The surface alkaline site densities of commercial MgO and MgO carriers prepared using the 4MgCO_3_·Mg(OH)_2_·4H_2_O precursor were compared by a CO_2_-TPD test (Figure 2b) combined with the N_2_ adsorption and desorption test. The weakly and strongly basic site densities of 5% Ru/MgO-DP were 3.7 and 8.8 μmol m^−2^, respectively, whereas the basic site concentrations of 5% Ru/c-MgO-DP prepared from homemade MgO carriers were 22 and 49 μmol m^−2^, respectively. The catalysts with a greater density of basic sites had a higher ammonia conversion at 450 °C under 30,000 mL g_cat_^−1^ h^−1^, reaching 80.6% for Ru/c-MgO and only 56.5% for Ru/MgO. Therefore, in combination with the above two works, in addition to the need for a suitable method of Ru nanoparticle loading, the creation of basic sites in the catalyst carrier is also important to facilitate ammonia decomposition. Jia et al. [64] investigated the catalytic process of Ru/MgO by using in situ DRIFTS as shown in Figure 2c,d. At 50 °C, ammonia can be rapidly adsorbed onto the catalyst, and as the temperature increased, obvious vibrational peaks of Ru-H and NH_2_ appeared, and as the temperature continued to increase, it was found that the intensity of the Ru-H was weakened but the intensity of the NH_2_ peaks was almost unchanged, which indicated that there was a gradual de-adsorption of H_2_ but that higher temperatures were still needed for the decomposition and desorption of NH_2_.

**Figure 3:**
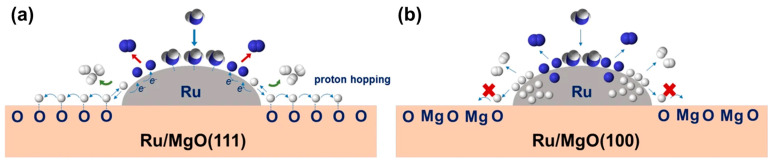
Mechanism illustration of ammonia decomposition on Ru/MgO(111) and Ru/MgO(100). The different behaviors of hydrogen hopping in (**a**) Ru/MgO(111) and (**b**) Ru/MgO(100) [50].

Tsang et al. [50] designed magnesium oxide-loaded Ru catalysts with different crystalline facets and investigated the conformational relationship of Ru on different facets in detail, in which the layer of oxygen atoms on the crystalline facets of MgO (111) could stabilize Ru as single atoms on the carrier. At the same time, catalytic effects similar to those at the B5 site can occur with neighboring Ru single atoms. DFT calculations show that the two Ru atoms on the MgO carrier can each adsorb NH_3_ molecules and then progressively undergo N–H bond-breaking dissociation with the MgO(111)-loaded Ru having the lowest energy barrier required for N-H bond breaking. In addition, during the transfer of H atoms to the surface of the MgO carrier, the energy barriers to be overcome on the surfaces of MgO (100) and MgO (110) are 0.96 eV and 1.8 eV, respectively, whereas the transfer of dissociated hydrogen atoms from the Ru atoms to the surface of the MgO (111) crystals does not require the overcoming of the energy barriers. Unlike the surface structure of MgO (100) and MgO (110), the hydrogen atoms transferred to the crystal surface of MgO (111) will gradually overflow outward due to the atomic layer of O atoms on the surface of MgO (111) as shown in Figure 3, which avoids the problem of catalyst poisoning caused by the aggregation of hydrogen atoms in the vicinity of Ru at the catalytic site. From the results, the ammonia conversion was 100% for Ru/MgO (111) but only about 70% and 50% for Ru/MgO (110) and Ru/MgO (100) at 450 °C under 30,000 mL g_cat_^−1^ h^−1^.

### 2.3. Rare Earth Oxide Support

In addition to their good chemical and thermal stability, rare earth metal oxides can be altered to regulate the acidity or alkalinity of the oxides, while the unique 4f electron layer of the rare earth metals allows them to act as electron acceptors or donors, which are capable of facilitating the transfer of electrons on the surface of the oxide carriers [65]. Eguchi and Muroyama et al. [66] have shown that the application of rare earth oxides as carrier materials in nickel-based catalysts has significant advantages. The activity of these catalysts was evaluated by loading Ni on different rare earth oxides (Y_2_O_3_, La_2_O_3_, CeO_2_, Sm_2_O_3,_ and Gd_2_O_3_) and by performance testing for ammonia decomposition. Among them, Ni/Y_2_O_3_ showed the best performance. To further understand the excellent performance of Ni/Y_2_O_3_ catalysts, NH_3_-TPSR (temperature-programmed surface reaction) experiments were performed to study the desorption behavior of N_2_ and H_2_ on the catalyst surface. The experimental results show that hydrogen is more easily desorbed on the Ni/Y_2_O_3_ catalyst than that of Ni/Al_2_O_3_. This finding suggests that the hydrogen adsorption site on the Ni/Y_2_O_3_ catalyst may have a lower adsorption strength, which helps to reduce the accumulation of hydrogen on the catalyst surface, thus avoiding the “hydrogen poisoning” phenomenon, in which the hydrogen adsorption site is occupied, hindering the further adsorption and decomposition of ammonia. In addition, Ni/La2O3 [61], Ru/Y_2_O_3_ [67], Ru/CeO_2_ [56] and Ru/Pr_2_O_3_ [59] all exhibit excellent catalytic performance.

The preparation of highly active ammonia decomposition catalysts through different synthesis methods has been studied in detail. Zhang and Feng et al. [68] prepared four kinds of La_2_O_3_ carriers by citric acid complexation, KOH-K_2_CO_3_ coprecipitation, pyrolysis, and the urea method, and they prepared Ru-based catalysts by the impregnation method. The influence of these catalysts on the decomposition reaction of NH_3_ was investigated. The La_2_O_3_ catalysts were more excellent in catalytic performance after citric acid complexation and then calcined at a high temperature of 700 °C. The authors attribute this to the fact that the high-temperature calcined La_2_O_3_ was able to make the Ru nanoparticles highly dispersed, and at the same time, it made it difficult for Ru to be sintered during the high-temperature catalytic process due to spatial isolation.

**Figure 4:**
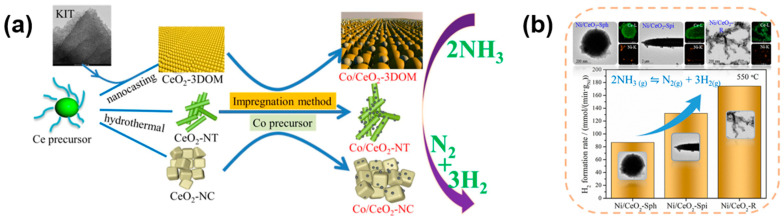
(**a**) Schematic diagram to illustrate the CeO_2_ with different morphology-supported Co catalysts for ammonia decomposition to hydrogen [52]. (**b**) Three different morphologies of CeO_2_ as a carrier for Ni/CeO_2_ catalysts and the corresponding catalytic properties for ammonia decomposition [54].

It is also crucial for the preparation of efficient catalyst carriers by adjusting the morphology of the catalyst carriers and thus their surface structural properties. Zhang et al. [52] prepared CeO_2_ with a three-dimensional ordered mesoporous structure, nanotube structure, and nanocubes structure, as shown in Figure 4a. The catalyst after loading Co, Co/CeO_2_-3DOM, had the most excellent catalytic performance for ammonia decomposition, which was completely decomposed at 600 °C and 6000 mL g_cat_^−1^ h^−1^. The CO_2_-TPD test showed that Co/CeO_2_-3DOM was more basic, which was significantly stronger than the other two catalysts. And the XPS test revealed that CeO_2_-3DOM as a catalyst carrier had the highest proportion of oxygen vacancies, indicating that the porous structure of the carrier had more unsaturated metal oxide sites. The H_2_-TPR test of the catalyst revealed that the reduction temperature of Co_3_O_4_ in Co/CeO_2_-3DOM was much higher than that of the other two morphologies of Co/CeO_2_, which indicated that there was a stronger interaction between Ce-O-Co and avoided the sintering of the Co-active site, and meanwhile, there was a reduction peak at 376 °C for CeO_2_-3DOM, which indicated that during the high-temperature decomposition of ammonia, the part of the generated hydrogen reduces the O in CeO_2_, causing O vacancy defects on the catalyst carrier surface, which makes it easy to form more surface active centers. After that, Jiang and Luo et al. [54] prepared CeO_2_ in rod, sphere, and spindle shapes by morphology modulation, which resulted in different sizes as well as dispersions of the loaded Ni nanoparticles due to the different morphologies. Among them, rod-shaped CeO_2_ has a larger specific surface area and smaller nickel nanoparticles compared to the other two forms of CeO_2_-loaded nickel nanoparticles, and it was found that rod-shaped CeO_2_ as a carrier can adsorb more NH_3_ by the NH_3_-TPD test, indicating that the rod-shaped CeO_2_ has more indicative acidic sites. In addition, the results of N_2_-TPD showed that Ni/CeO_2_-R has a lower N_2_ desorption temperature, indicating that there is more electron transfer between the rod-shaped CeO_2_ and Ni to desorb N_2_ from the metal active center, which improves the kinetic performance of ammonia decomposition and therefore has the best catalytic activity. In addition, the spindle morphology exposed more (110) crystalline surfaces of CeO_2_, which gave Ni/CeO_2_-Spi more strongly basic sites and thus favored the transfer of electrons to the Ni particles and promoted the binding desorption of N atoms (Figure 4b).

In addition to the morphology of the catalyst and the construction of the basic sites, the binding and desorption of N atoms on the catalytic sites is facilitated by the addition of promoters to provide additional electrons [35,39,69,70,71]. Nagaoka et al. [72] explored the effects of alkali metal oxides, alkaline-earth metal oxides, and rare-earth metal oxides on ammonia decomposition by introducing these promoters into Ru/Pr_6_O_11_ catalysts. Among them, the doped Cs_2_O was able to provide more electrons to promote the binding and dissociation of N atoms due to the strongest basicity of Cs_2_O. In addition, by adjusting the loading order of Ru and Cs_2_O, it was found that Ru loading on Cs_2_O/Pr_6_O_11_ increased the conversion of NH_3_ from 76% to 93% at 350 °C under 3000 mL g_cat_^−1^ h^−1^. This is attributed to the fact that the loading of Ru after Cs_2_O avoids the disadvantage of the reduction in catalytic sites due to the covering of Ru by Cs_2_O, while it can still play the role of Cs_2_O promoter, which further improves the catalytic effect.

The ammonia decomposition conversion and hydrogen production rates corresponding to single metal oxide-based catalysts are summarized in Table 1. Suitable metal oxide carriers were selected, and the interaction between active metal nanoparticles and carriers was improved by various synthesis methods. The catalytic activity of single metal oxides was mainly related to the size of metal nanoparticles, the specific surface area of the catalyst, and the electronic structure of the catalyst surface. The performance of the catalysts can be further improved by adjusting the acidity and alkalinity of the carrier surface as well as the differences in the crystalline surface effects of the carriers to improve the dissociation behavior of nitrogen and hydrogen during the catalytic process.

## 3. Mixed Metal Oxide Supports

### 3.1. Regulating the Size of Active Site Particles

**Figure 5:**
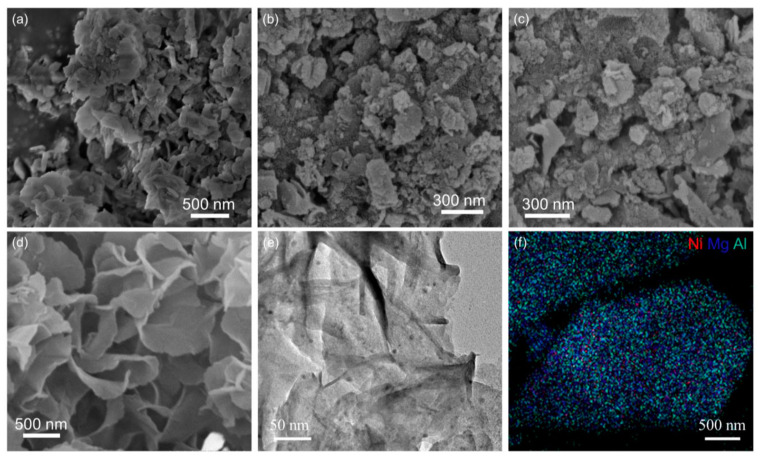
SEM images of the reduced (**a**) Ni/MgAl_2_O_4_-MM, (**b**) Ni/MgAl_2_O_4_-SG-1, (**c**) Ni/MgAl_2_O_4_-SG-2, and (**d**) Ni/MgAl_2_O_4_-LDH, and (**e**) TEM image, (**f**) TEM mapping of reduced Ni/MgAl_2_O_4_-LDH [76].

Metal oxide supports improve the interaction with metal nanoparticles by doping heteroatoms and modulating the properties of the metal oxides themselves, changing the size of the metal nanoparticles. In this way, the adsorption of ammonia and the desorption rate of nitrogen and hydrogen can be regulated. Qiu et al. [69] used Ca-Al layered double hydroxides (LDH) as a catalyst carrier and RuCl_3_ as a precursor for Ru catalytic sites, and they prepared the Ru/CaAlO_x_ catalysts by two methods, respectively, dispersing Ru on the lower surface of CaAlO_x_ by wet-chemical impregnation and by a mixed deposition of Ca, Ru, and Al ions followed by a high-temperature heat treatment to achieve the surface remodeling of LDH Ru/CaAlO_x_ catalysts. TEM characterization of the catalysts and size counting of Ru nanoparticles revealed that the size distribution of Ru nanoparticles obtained with the aid of LDH surface reconstruction (Ru/CaAlO_x_-w) was more homogeneous. Moreover, the ammonia conversion was close to 100% at 1.5 wt% Ru loading at 600 °C under 6000 mL g_cat_^−1^ h^−1^. In addition, Liu et al. [75] also used Ca-Al LDH as a catalyst carrier to control the size of Ni nanoparticles by introducing different Ni precursors (NiCl_2_·6H_2_O, Ni(NO_3_)_2_·6H_2_O) and changing the doping ratio of Ni. The analysis of TEM and the statistics on the size of Ni nanoparticles revealed that when Ni(NO_3_)_2_·6H_2_O was used as the precursor, the particle size of Ni obtained was smaller. Therefore, with the same mass fraction of Ni loading, the Ni/CaAlO_x_(NO_3_^−^) one has more active sites, and its conversion of ammonia reaches 89.1% at 500 °C under 6000 mL g_cat_^−1^ h^−1^, while that of Ni/CaAlO_x_(Cl^-^) is less than 50%. The Ni/MgAl_2_O_4_ system was employed as a catalyst for ammonia decomposition by Xiao et al. [76]. They prepared MgAl_2_O_4_ using mechanical mixing, sol–gel, and hydrothermal methods, respectively, and named MgAl_2_O_4_-MM, MgAl_2_O_4_-SG, and MgAl_2_O_4_-LDH. Ni was loaded onto MgAl_2_O_4_ using conventional wet chemical impregnation, and it can be seen by SEM and TEM (Figure 5) that the Ni/MgAl_2_O_4_-LDH was a layered structure and the Ni nanoparticles in the catalyst were highly dispersed, which helped to improve the ammonia decomposition activity of the catalyst and alleviate the phenomenon of H_2_ poisoning. Meanwhile, the H_2_-TPR test results revealed that the Ni/MgAl_2_O_4_-LDH with a layered structure had the highest reduction peak temperature, which indicated its good thermal stability, and thus the ammonia conversion could be stabilized at 88.7% and 100% at 600 °C and 650 °C under 30,000 mL g_cat_^−1^ h^−1^, respectively. Therefore, it is important to control the size of the active metal sites to expose more B5 active sites, and factors in this regard have been explored in previous work [63].

### 3.2. Basic Sites on the Surface of Catalyst Supports

**Figure 6:**
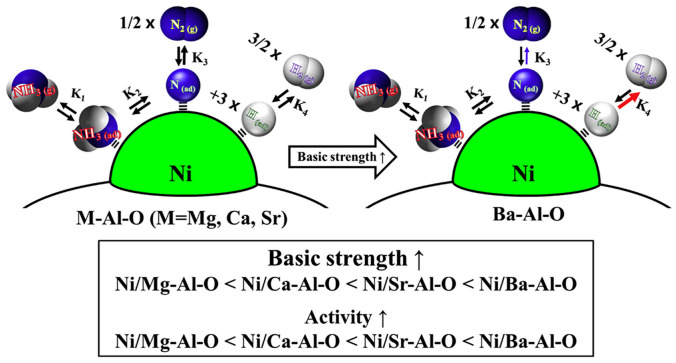
Schematic representation of catalyst basic site strength and catalytic activity mechanism [78].

Ji et al. [77] also used LDH as precursors to regulate the surface basicity of the catalyst carriers as well as the particle size of Ni at the catalytic sites by adjusting the ratios of Ni/Mg and Mg/Al. When the ratio of Mg/Ni was adjusted, there were more basic sites of the catalyst with the increase in the Mg element; meanwhile, according to the results of H_2_-TPR, the higher the percentage of the Mg element, the higher the reduction temperature of the catalyst. This suggests that the increase in the number of electrons on the surface of the catalyst through the doping of Mg improves the electron transfer from the carrier surface to the active metal center, thus improving the stability of the catalyst. During the process of ammonia decomposition, Ni_0.12_Mg_1.09_Al_0.3_O_n_ has the largest hydrogen–hydrogen transition frequency (TOF) of 8.1 s^−1^. Therefore, by adjusting the surface acidity and alkalinity of the carrier catalysts as well as the interactions between the carriers and the catalyst particles, it helps to improve the performance of ammonia decomposition. Eguchi et al. [78] modulated the surface basic density of the Al_2_O_3_ carrier by individually doping different alkaline earth metals (Mg, Ca, Sr, or Ba) into Al_2_O_3_. The difference in basicity between the different alkaline earth metals resulted in different surface basic densities of the Al_2_O_3_ carriers, and consequently, there was a difference in the performance of the catalysts, as shown in Figure 6. The CO_2_-TPD test of the alkaline earth metal-modified catalyst (Ni/AM-Al-O) revealed that Ni/Ba-Al-O contained a high number of strongly basic sites. Therefore, during the ammonia decomposition test, Ni/Ba-Al-O embodied the best catalytic performance with a conversion rate of 90.3% at 550 °C under 6000 mL g_cat_^−1^ h^−1^. Zhang [79], Wang [39], and Chae [80] et al. also modified the surface acidity and alkalinity of the oxide carriers by elemental doping, and the performance of the catalysts was significantly enhanced, effectively improving the performance of ammonia decomposition.

Perovskite oxides are a class of materials with a specific crystal structure, which are regarded as good catalyst carriers for ammonia decomposition due to their structural diversity, high specific surface area, and adjustable acid–base properties [81,82]. A typical catalyst loaded with Ru nanoparticles using ABO_3_ perovskite oxide (LaAlO_3_) as a carrier was reported by Luo and Zhou et al. [83]. By adjusting the cationic substitution of Sr^2+^ for La^3+^, the electronic properties of the carrier material were modulated, thus affecting the electronic state of the Ru active center. The adjustment of the ratio of Sr and La resulted in different electronic structures on the surface of the catalyst carriers, showing different catalytic effects. With the increase in the ratio of Sr/La, the catalytic activity showed a volcano-type change trend, in which Ru/La_0.8_Sr_0.2_AlO_3_ presented the best catalytic performance, with the conversion rate of ammonia reaching 71.6% at 500 °C under 30,000 mL g_cat_^−1^ h^−1^, and it was able to keep the catalytic activity without decaying at 550 °C for 70 h. The results showed that the catalytic activity of the Ru/La_0.8_Sr_0.2_AlO_3_ was very good with the catalyst being able to maintain its catalytic activity without decaying for 70 h. The in situ spectroscopic studies and NH_3_-TPD analyses showed that the metal Ru was in an electron-rich state on the La_0.8_Sr_0.2_AlO_3_ carrier, which facilitated the N atom associative desorption and accelerated the NH_3_ decomposition reaction. The H_2_-TPR test also showed that the doping of Sr^2+^ could increase the reduction temperature of the catalyst, indicating a stronger interaction between Ru and La_1-__x_Sr_x_AlO_3_, which effectively avoided the agglomeration of Ru nanoparticles during the prolonged catalytic process under high-temperature conditions and improved the stability of the catalyst.

### 3.3. Oxygen Vacancy Defect Sites on Catalyst Carrier Surfaces

**Figure 7:**
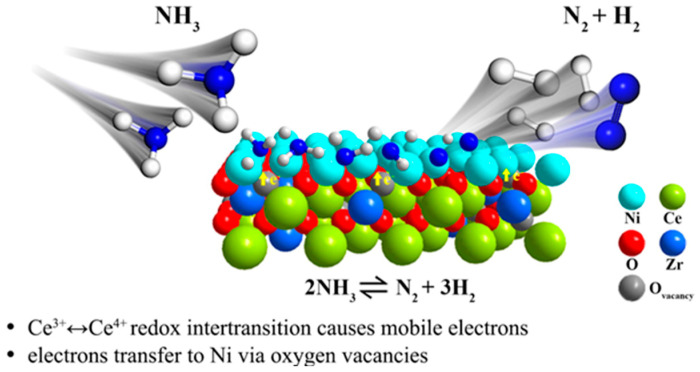
Schematic that depicts the reaction mechanism of NH_3_ decomposition over Ni/Ce_1_-_x_Zr_x_O_2_ catalysts [84].

Surface oxygen vacancies can be used as active sites to increase the number of active centers on the catalyst surface as well as to change the electronic structure of the material and promote charge transfer, and an appropriate amount of oxygen vacancies can improve the thermal and chemical stability of the material. Jiang et al. [84] produced oxygen vacancies in the synthesized Ce_1-__x_Zr_x_O_2_ by doping CeO_2_ with different concentrations of Zr and utilizing the size difference between Zr and Ce atoms, as shown in Figure 7. The Raman spectroscopy test of the material revealed that the concentration of oxygen vacancies in Ce_1-__x_Zr_x_O_2_ showed a volcano-type change of increasing and then decreasing with the increase in the doping ratio of Zr element, which indicated that there is a limit to the capacity of oxygen vacancies in CeO_2_, and the ratio of Ce^3+^/Ce^4+^ also increases with the concentration of oxygen vacancies. Overall, it indicates that the number of electrons transferred between the carrier and the metal active center varies. The direction of electron transfer is shown in Figure 7. Meanwhile, according to the test results of ammonia decomposition, it was found that the trend of the catalyst for ammonia conversion was consistent with the trend of the oxygen vacancy concentration. Therefore, it is shown that the presence of oxygen vacancies in the catalyst has a significant enhancement on the catalytic process. Based on the NH_3_-TPSR test results of the catalysts, it was found that for N_2_ formation and desorption, the doped Ni/Ce_1-__x_Zr_x_O_2_ catalysts were more effective than Ni/CeO_2_, exhibiting a temperature reduction of about 53 °C. Thus, it can be determined that the oxygen vacancies in CeO_2_ can provide moving electrons and electronically promote the cleavage of metal-N bonds, thus accelerating the N_2_ conjugation desorption. Li and Liu et al. [40] similarly used Ni/Ce_0.8_Zr_0.2_O_2_ as a catalyst, based on which the Al element was further introduced to form an Al-Ce_0.8_Zr_0.2_O_2_ carrier. The characterization and results showed that the introduction of Al as a secondary dopant increased the specific surface area and surface oxygen defects of cerium oxide, enhanced the dispersion of nickel metal and metal–carrier interaction, and thus improved the catalytic performance of ammonia decomposition. The oxygen defect sites on the surface facilitate the recombination of dissociated hydrogen atoms on its surface to form hydrogen molecules, as well as the recombination and desorption of N_2_, which restores its surface active sites.

### 3.4. Active Site In Situ Generation

**Figure 8:**
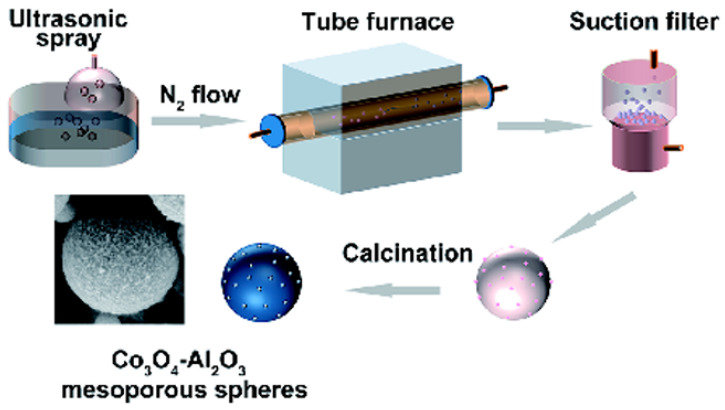
Schematic diagram of the preparation process with the AASA for Co_3_O_4_-Al_2_O_3_ mesoporous spheres [44].

In addition to improving the interaction between the metal nanoparticles and the carriers, it is also an effective method to improve the stability as well as the sintering resistance of the metal nanoparticles by doping the active metals into the carriers in the form of oxides, which are reduced in situ to active metal sites by H_2_ generated during ammonia decomposition. Chunjiang Jia’s group [44] prepared a mesoporous Co_3_O_4_-Al_2_O_3_ catalyst by aerosol-assisted self-assembly development (Figure 8). The specific surface area of the catalyst was tuned by adjusting the ratio of Co/Al to increase the number of active sites. Among them, 90CoAl has the largest specific surface area and at the same time has the optimal catalytic performance. The conversion of ammonia reached 90% when at 550 °C and 18,000 mL g_cat_^−1^ h^−1^. The in situ XRD test of the catalysts under H_2_-TPR conditions revealed that with the increase in temperature, Co_3_O_4_ was first reduced to CoO, and then it continued to be reduced to metal Co. When Co/Al was larger, the reduction temperature of Co_3_O_4_ was lower, which allowed the ammonia to be converted at a lower temperature at the metal active sites. In addition to this, Jia et al. developed a series of metal oxide catalysts for in situ reduction, such as NiO-Al_2_O_3_ [85], CoAl_2_O_4_ [86], Ni-Ce-Al-O [87] and Co_a_Sm_b_O_x_ [88].

Unlike monometallic oxides, mixed-metal oxides can be doped with heteroatoms to modulate the acid–base, electronic structure, and oxygen-deficient sites of the carrier oxides, thereby increasing the activity of the catalyst. The ammonia decomposition conversion and hydrogen production rates corresponding to mixed-metal oxide-based catalysts are summarized in Table 2. The selection of different alkali metal and alkaline earth metal catalysts can effectively increase the basic sites on the surface of carrier oxides; secondly, considering the difference in atomic radii between the heteroatoms and the metal atoms of the carrier oxides, some oxygen vacancy defects will appear in the regular arrangement of the crystals. Therefore, in addition to the intrinsic activity of the metal catalytic sites, the kinetic performance of the ammonia decomposition process can be enhanced by modulating the structure as well as the properties of the carriers, which in turn improves the ammonia adsorption as well as the desorption performance of nitrogen and hydrogen, promoting the rapid decomposition of ammonia at lower temperatures.

## 4. Metal Oxides Based on Metal–Organic Frameworks (MOFs)

**Figure 9:**
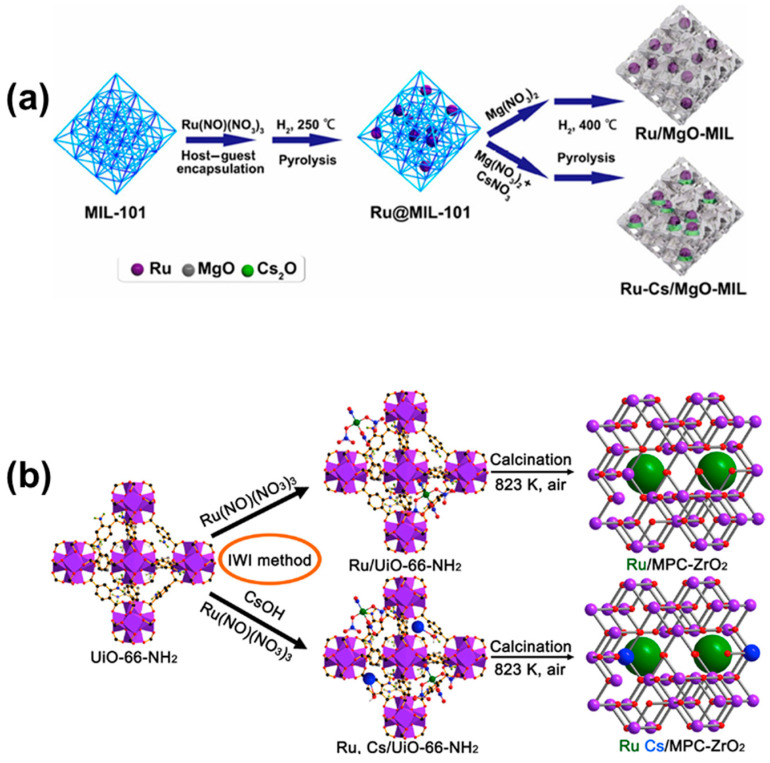
(**a**) Schematic synthetic protocols of MIL-101-confined Ru clusters [92]. (**b**) Schematic illustration for preparing Ru and Ru/Cs catalysts on mesoporous zirconia using a combination of incipient wetness impregnation (IWI) and calcination approach [74].

MOFs are a type of porous material consisting of metal ions or metal clusters connected with organic ligands through strong coordination bonds. They are structurally diverse, and materials with specific properties can be designed by changing the metal ions, organic ligands, or their combinations. In addition, MOFs have a very high specific surface area and contain a large number of active sites. By adjusting the conditions of high-temperature calcination, metal oxides with regular morphology and a large number of pore structures can be obtained. In addition, MOFs with multi-metal components can be synthesized by changing metal ions, and further multi-metal doped metal oxides can be obtained. Chen and Zhu et al. [92] used MIL-101 as a template, impregnated Ru(NO)(NO_3_)_3_ into the skeleton of MIL-101, and reduced it to Ru nanoparticles by hydrogen. Then, they further impregnated CsNO_3_ and Mg(NO_3_)_2_ into the skeleton of MIL-101 and then prepared the catalysts with the porous skeleton of MIL-101 derivatives by the high-temperature calcination of the Ru-Cs/MgO-MIL catalyst (Figure 9a). The addition of CsO and MgO provides additional electrons for Ru nanoparticles, and the porous structure of the catalyst can provide more active sites for the decomposition of ammonia. It can also effectively avoid the sintering of Ru nanoparticles in the decomposition of ammonia at high temperatures, ensuring the stability of the catalyst structure. EXAFS spectra results show that Ru is present in the catalyst with Ru-Ru and Ru-O bonds, indicating that Ru-O forms strong interactions that contribute to the stability of Ru nanoparticles on the catalyst surface. The ammonia decomposition performance test found that the conversion rate of ammonia reached 100% when at 450 °C under 15,000 mL g_cat_^−1^ h^−1^. In addition, Murciano et al. [74] used UiO-66(Zr)-NH_2_ as the precursor of the catalyst carrier: firstly, Ru(NO)(NO_3_)_3_ and CsOH were impregnated into the pores of UiO-66(Zr)-NH_2_ and then calcined at high temperature at 550 °C to calcine Ru, Cs/UiO-66-NH_2_ as a catalyst carrier with ZrO_2_ as a carrier catalyst (Ru-Cs/MPC-ZrO_2_) (Figure 9b). By adjusting the ratio of Ru and Cs, the obtained catalyst 5 wt% Ru 5 wt% Cs/MPC-ZrO_2_ achieved 66.7% conversion of ammonia at 400 °C under 6000 mL g_cat_^−1^ h^−1^.

The ammonia decomposition conversion corresponding to MOF-based catalysts is summarized in Table 3. However, at present, there is still less work on the synthesis of catalysts for hydrogen production from ammonia decomposition using MOF materials as precursors. Based on the excellent structural features of MOF materials themselves, when MOFs are used as templates or precursors, they can be transformed into metal/carbon-based porous materials that are much more stable than the pristine MOFs. In addition, to a large extent, they are capable of inheriting the properties of the pristine MOFs, such as the large specific surface area, the component diversity and dispersion, customizable porosity, and tunable metal components, etc., which gives them great potential in high-temperature catalytic reactions. It can be seen that MOFs have great potential for application as catalysts.

## 5. Conclusions and Perspectives

Loaded metal oxide catalysts are a class of multiphase catalysts widely used in industrial production and scientific research, and they have been recognized by a large number of studies as one of the most effective catalysts for ammonia decomposition. We review the recent research work by loading metals on metal oxides to NH_3_ decomposed. The metal–metal oxide interfacial effect is an important factor affecting the performance of loaded metal oxide catalysts. It has been found through many studies that the particle size of the metal active sites, the number of basic sites of the metal oxide carriers, and the atomic configurations of the metal oxide surfaces have an impact on the catalytic activity. By adjusting the particle size of metal active sites, more B5 active sites can be exposed. In addition, increasing the alkalinity of the metal oxide carrier and adjusting the O-atom configuration of the metal oxide carrier accelerated the desorption of N_2_ and H_2_, improving the kinetic performance of ammonia decomposition.

At present, the most effective catalysts for ammonia decomposition are those based on Ru, doped with K, Ba and Cs, and loaded on a variety of carbon carriers and metal oxides. Nevertheless, the study of ammonia decomposition catalysts is still in its infancy, and there are several shortcomings that need to be addressed. Firstly, the kinetics and rate-limiting steps of the ammonia decomposition reaction have yet to be fully elucidated despite the progress made in existing studies. Secondly, while some effective catalysts for ammonia decomposition have been developed, such as Ru-based catalysts, they are frequently costly. Furthermore, while non-precious metal catalysts are less expensive, their activity and stability remain considerably inferior to those of precious metal catalysts. It is therefore necessary to modulate the morphology, size and surface electronic structure of the catalysts in order to improve their performance. This can be achieved by developing new synthetic methods such as templating, atomic layer deposition and electrochemical deposition and by optimizing the synthetic conditions according to the currently known ammonia decomposition reaction mechanism. It is expected that loaded catalysts with excellent catalytic performance will be developed, which will provide an important material science basis for the future development of efficient and green ammonia energy systems.

## Figures and Tables

**Table 1:** Comparison of catalytic activity over single metal oxide catalysts for ammonia decomposition.

Catalyst	Active Metal Content/wt %	GHSV /mL g_cat_^−1^ h^−1^	T_a_/°C	NH_3_ Conversion/%	T_b_/°C	H_2_ Formation Rate	Ref.
7.6Co/Al_2_O_3_-carb	7.6 (Co)	6000	550	100	500	156.4 ^a^	[43]
Co-Ni/Al_2_O_3_	4.72 (Co) 4.91 (Ni)	30,000	700	100	-	-	[46]
Ni@Al_2_O_3_	25 (Ni)	24,000	600	93.9	450	7.8 ^b^	[42]
Ru/κ-Al_2_O_3_(C300)	1.3 (Ru)	60,000	450	80	400	42 ^a^	[63]
Co@Al_2_O_3_-550	8 (Co)	15,000	550	70	-	-	[73]
K-5%Ru/MgO-DP	3.5 (Ru)	36,000	475	100	475	40.2 ^b^	[48]
Ru/c-MgO	4.7 (Ru)	30,000	450	80.6	450	27 ^b^	[49]
Ru/MgO	1.7 (Ru)	60,000	450	83	450	36 ^b^	[74]
Ru/MgO(111)	3.1 (Ru)	60,000	450	82.2	450	106.6 ^a^	[50]
Ni/La_2_O_3_	40 (Ni)	6000	550	78.9	-	-	[61]
Ru/Cs_2_O/Pr_6_O_11_	5 (Ru) 2.5 (Cs)	3000	350	93	-	-	[72]
Ni/Y_2_O_3_	40 (Ni)	6000	500	62.2	-	-	[66]
Ru/La_2_O_3_-700-i	4.8 (Ru)	18,000	525	90.7	450	11.7 ^b^	[68]
Co/CeO_2_-3DOM	5 (Co)	6000	500	62	500	4.2 ^b^	[52]
Cs-Ru/CeO_2_	8.92 (Ru) 2.82 (Cs)	2000	300	80	-	-	[51]
Ru/Y_2_O_3_	5 (Ru)	30,000	460	97	450	30.81 ^a^	[67]
Ru/Sm_2_O_3_-p	3.8 (Ru)	30,000	450	84.6	450	25.9 ^b^	[60]
Ni-Ru/CeO_2_	2.39 (Ni) 0.35 (Ru)	3660	450	88.7	-	-	[55]
Ru/Pr_2_O_3_	5 (Ru)	30,000	450	68.2	450	20.9 ^b^	[59]
Ni/CeO_2_-R	8.41 (Ni)	30,000	650	88	550	10.46 ^a^	[54]

T_a_ Temperature corresponding to ammonia conversion. T_b_ Temperature corresponding to H_2_ formation rate. ^a^ mol_H2_ g_M_^−1^ h^−1^. ^b^ mmol_H2_ g_cat._^−1^ min^−1^.

**Table 2:** Comparison of catalytic activity over mixed-metal oxide catalysts for ammonia decomposition.

Catalyst	Active Metal Content	GHSV /mL g_cat_^−1^ h^−1^	T_a_/°C	NH_3_ Conversion/%	T_b_/°C	H_2_ Formation Rate	Ref.
La-modified Ni/Al_2_O_3_	19 (Ni) ^a^	6000	550	92	-	-	[47]
(Co,Al)_2_O_4_	90/10 (Co/Al) ^b^	18,000	600	100	-	-	[86]
90CoAlO_x_	90/10 (Co/Al) ^b^	36,000	600	100	-	-	[85]
Ni_0.5_Ce_0.1_Al_0.4_O_x_	5/1/4 (Ni/Ce/Al) ^b^	18,000	500	90	400	109 ^d^	[87]
Ni/Zr-doped Al_2_O_3_	20 (Ni) ^a^	7500	550	79.8	600	6.6 ^d^	[45]
Ru/CaAlO_x_	3.5 (Ru) ^a^	6000	500	98.2	-	-	[69]
20Co/La-MgO(5)	20 (Co) ^a^	124,000	550	62	550	91 ^d^	[89]
Ni_1_Co_9_/CZY	1 (Ni) ^a^ 9 (Co)	6000	600	100	-	-	[38]
Cs-Ru/Ba-ZrO_2_	3 (Ru) ^a^	30,000	450	37.8	450	12.7 ^d^	[39]
Ni/Al-Ce_0.8_Zr_0.2_O_2_	8 (Ni) ^a^	9000	640	100	-	-	[40]
Ni/MgO-La_2_O_3_	5 (Ni) ^a^	30,000	650	54	650	1258 ^c^	[79]
Ni/Ba-Al-O	20 (Ni) ^a^	6000	550	95	-	-	[78]
Ni/Ce_0.85_Zr_0.15_O_2_	9.7 (Ni) ^a^	30,000	600	88	-	-	[84]
0.5%Ba/CoCe (80/20)	41.4 (Co) ^a^ 23.5 (Ce) 0.45 (Ba)	9000	450	80	-	-	[71]
Co_0.7_Sm_0.3_O_x_	7/3 (Co/Sm) ^b^	23,000	550	100	500	97.2 ^d^	[88]
Ru/La_0.33_Ce_0.67_	1.8(Ru) ^a^	6000	400	90	400	6.2 ^d^	[80]
20Ni/Al_1_Ce_0.05_O_x_	19.3 (Ni) ^a^	6000	500	100	500	5.7 ^d^	[90]
K-CoNi/MgO-CeO_2_-SrO	40 (Co) ^a^ 10 (Ni) 2/1/12 (Mg/Ce/Sr) ^b^	18,000	475	93	450	8.26 ^d^	[91]
Ni/CaAlO_x_(NO_3_^−^)-R	24.7 (Ni) ^a^	6000	500	89.1	-	-	[75]
Ni/MgAl_2_O_4_-LDH	4.49 (Ni) ^a^	30,000	650	100	600	29.71 ^d^	[76]
Ru/La_0.8_Sr_0.2_AlO_3_	2.55 (Ru) ^a^	30,000	500	71.6	500	56.46 ^c^	[83]
La_0.1_Mg_0.9_NiO_3_	1/9/10 (La/Mg/Ni) ^b^	75,000	350	72.3	350	3 ^d^	[82]

^a^ wt%. ^b^ Ratio of atomic weights of metals. T_a_ Temperature corresponding to ammonia conversion. T_b_ Temperature corresponding to H_2_ formation rate. ^c^ mol_H2_ g_M_^−1^ h^−1^. ^d^ mmol_H2_ g_cat._^−1^ min^−1^.

**Table 3:** Comparison of catalytic activity over MOF-based catalysts for ammonia decomposition.

Catalyst	MOF Precursor	Active Metal Content/wt%	GHSV /mL g_cat_^−1^ h^−1^	T/°C	NH_3_ Conversion/%	Ref.
Ru-Cs/MPC-ZrO_2_	UiO-66-NH_2_	5 (Ru) 5 (Cs)	6000	400	66.7	[74]
Ru-Cs/MgO-MIL	MIL-101(Cr)	4 (Ru)	15,000	450	100	[92]
Fe-BTC@400	Fe-BTC	34.7 (Fe)	6000	500	73.8	[93]
